# Assessment of Constraints of Artificial Insemination Service in Smallholder Dairy Cattle Keepers in Kacha Bira District of Southern Ethiopia

**DOI:** 10.1155/2023/6512010

**Published:** 2023-03-31

**Authors:** Mesfin Mathewos, Habtamu Endale, Mulugeta Tesfahun, Dembelo Tiele, Remedan Bukero

**Affiliations:** ^1^Wachemo University, School of Veterinary Medicine, Hossana, Ethiopia; ^2^Wolaita Sodo University, School of Veterinary Medicine, Wolaita Sodo, Ethiopia

## Abstract

Artificial insemination (AI) is among the most effective reproductive biotechnologies that afford widespread propagation of genes carried by superior males. A cross-sectional study followed by a simple random sampling technique was conducted from December 2021 to May 2022 to assess the constraints of artificial insemination (AI) provision in and around Kacha Bira district, Southern Ethiopia, using a structured questionnaire. A total of 200 respondents were surveyed accordingly. In this study, the education level of farmers revealed no statistically significant difference (*P* > 0.05) with the identification of time of insemination. Conception failure (62.5%), unavailability of artificial insemination technicians (7.5%), dystocia (3.5%), and both conception failure and unavailability of artificial insemination technicians (4.5%) were found to be the major constraints of AI service in the study area according to dairy cattle owners' response and revealed a statistically significant difference (*P* < 0.05) with AI service. Although statistically significant (*P* < 0.05), differences in AI service interruptions during both regular working hours and weekends and holidays were also observed. Among total respondents, 20.5% of dairy cattle owners got AI service at right time, but 79.5% of them used it at the wrong time. Regarding inbreeding problems, 77.5% of dairy cattle owners responded that there was no inbreeding problem and the remaining 22.5% of farmers indicated presence of inbreeding problem of which 10.5% and 10% had a perception that local breeds had low milk production and low genetic improvement than exotic breeds, respectively. On the other hand, 11.5% of dairy cattle owners responded that local breeds have a similar level of disease resistance to that of exotic breeds (11%). 48.5% of dairy farmers reported that bellowing is the most frequent sign that they used to detect heat followed by vulval discharge (23%) and mounting on other cows (10%). Majority (78.5%) of the dairy cattle owners interviewed were found to be not satisfied with the artificial insemination services. In general, different AI technicians and cattle and dairy cattle keeper-related factors constrain the AI service and its result in survey site. Therefore, smallholder dairy cattle owners should be trained sufficiently about the AI service strategies, usage, and proper management of dairy farms and the technical constraints should be avoided in order to provide AI service sufficiently.

## 1. Introduction

Compared to other African nations, Ethiopia has the biggest population of livestock [1]. It has one of the largest livestock populations in the world, including 59.5 million cattle which put the nation first in Africa and sixth in the world, 30.7 million sheep which put it third in Africa and tenth in the world, and 30.2 million goats which put it again third in Africa and eighth in the world [2]. About 85% of the people in Ethiopia are employed in agriculture, which mostly consists of crop and livestock production [3]. About 35–49% of Ethiopia's agricultural GDP and 16-17% of the country's foreign exchange profits are derived from the livestock [4].

There are an estimated 55.03 million cattle in Ethiopia's rural sedentary regions, of that 55.38% are female which consists of 6,675,466 dairy and 10,731,656 milking cows [5]. Local breeds account for 98.95%, those crossbred are 0.94%, and the remaining 0.11% are pure exotic breeds [6]. The total amount of milk produced for the 2015-16 fiscal year was estimated to be 3.06 billion litres, with an average lactation period of 6 months and an average daily milk production of 1.67 litres per cow [7].

This suggests that the total number of both exotic and hybrid female cattle produced through the crossbreeding work for many decades in the country is quite insignificant indicating unsuccessful crossbreeding work. This again suggests that Ethiopia needs to work hard on improving the work of productive and reproductive performance improvements of cattle through appropriate breeding and related activities [7].

By importing semen rather than live animals, artificial insemination (AI) technology maximises the utilization of outstanding males, the transmission of superior genetic material, the rate and effectiveness of genetic selection, and the introduction of new genetic material [8]. Because it is simple, economic, and successful, AI is the most important assisted reproductive technology in developing countries [9]. It is the most advantageous management technique available to cow producers, and it has been extensively employed to breed dairy cattle. It has also made bulls with high-quality genetic makeup broadly available to everyone [10].

Although artificial insemination, the most widely used and beneficial technology [11], has been employed in Ethiopia for more than 30 years, the effectiveness and impact of the practice have not been adequately documented [12]. However, AI service is getting weak and even declining due to inconsistent service delivery in the smallholder livestock production systems of Ethiopian highlands [13]. The problem is more aggravated by a lack of a recording system, estrous detection problems, wrong selection procedures, and poor management of AI bulls and skills of inseminators [14].

There was a limitation in AI service provision and its efficiency is poor in different parts of Ethiopia, thus it has not been successful to improve the reproductive performance of the dairy cattle and the country's diary sector [1, 15, 16]. However, there were no published works on the causes and challenges for the poor efficiency of AI service in this study site. Thus, the current study was aimed to assess the AI service delivery system in cattle and assess the associated risk factors and/or challenges of AI service in the area.

## 2. Materials and Methods

### 2.1. Study Area

This study had been conducted in the Kacha Bira district, Kembata Tembaro Zone, which is found in Southern Ethiopia. Kembata Tembaro Zone is one of the fourteen zones and four special districts in the SNNPR, and its capital is Durame. Kacha Bira district is located at 7°10′–7°34′N latitude and 37°58′–37°86′E longitude. The altitude of the district ranges from 1650 to 2450 meters above sea level. In terms of topography, the district has suitable land for agriculture except for some hilly areas. The annual rainfall varies from 900 to 1500 mm, and the main rainy season is from June to September. The annual mean temperature varies from 14 to 26°C [17]. The woreda (district) consists of 20 rural and 6 urban kebeles (peasant associations). The total human population of the district (in 2010) was 133,303. The total number of households is 18,605 where 15,238 are male and 3,367 are female. The total land area is 36,790 hectares; out of these, 21,875 hectares are suitable for agriculture. Small-level dairy production is common in the area, and they practice mainly semi-intensive management systems. Exotic, mainly Holstein Friesian, breed is common in the area, but local breeds are also farmed [17].

### 2.2. Study Population

The study population included smallholder dairy cattle owners who were randomly selected from five kebeles of the Kacha Bira district practicing AI in their farms.

### 2.3. Study Design

A cross-sectional type of study using a questionnaire survey was carried out from December 2021 to May 2022 to assess the constraints associated with artificial insemination (AI) service in and around Kacha Bira district, Southern Ethiopia. Data about the status of AI service in different kebele such as availability of AI service during weekends and holidays, access to inputs and AITs, knowledge of dairy cattle owners of the different signs of estrus, AI service problems identified by dairy cattle owners, and owners' satisfaction with AI services were recorded.

### 2.4. Sample Size Determination and Sampling Procedures

Study population was nominated by a simple random sampling technique from five kebeles selected based on the frequency of smallholder dairy cattle keepers according to the data obtained from the district's livestock and fishery office. The sample size for the survey was calculated according to the formula designed in [18], considering a standard error of 5% at 95% confidence levels. Thus, the number of respondents was determined from 643 smallholder dairy cattle owners found in the selected kebeles based on the formula given below:

(1) $$\begin{array}{c} n=\frac{n0}{\left(1+n0/Population\right)}\text{,} \end{array}$$

where population size = 643; *n*0 = required return sample size according to Cochran's formula = 118; and *n* is the total required sample.

Accordingly, the substitution of the values in the above formula gives the total required sample size of 100. However, considering the design effect, the sample size was doubled and a total of 200 smallholder dairy cattle keepers practicing AI on their farms were included in the study.

### 2.5. Data Collection

Face-to-face interview of the smallholder dairy cattle owners selected through simple random technique was used to conduct structured questionnaires to gather information on the status of AI services provided in the district, including delivery methods, consistency of service delivery, weekend service availability, daily accessibility of inseminators, and limitations associated with the service from the perspective of dairy cattle owners. Before going over to the actual questions, each respondent was introduced about the study goal.

### 2.6. Data Management and Statistical Analysis

The data collected during the study period were entered into Microsoft Excel, imported, and analyzed using STATA software version 13 (StataCorp., College Station, TX). Descriptive statistics like percentages and tables were used to express prevalence while Pearson's chi-square (*x*^2^) test was used to compare the association of association AI services with different risk factors.

## 3. Results

### 3.1. Assessment of AI Service in the Study Area

Among the 200 dairy cattle owners surveyed, 163 (81.5%) got AI service consistently, whereas 37 (18.5%) of the respondents were unable to do so. Of these 37 owners, 8 (4%) and 12 (6%) were unable to receive services due to a lack of AITs, 3 (1.5%) were unable to receive services because of a shortage of inputs, 3 (1.5%) were unable to receive services due to all reasons, and 11 (5.5%) were unable to receive services for unspecified reasons. In the AI service between the study kebeles, there was a statistically significant difference (*P* value <0.05) (Table 1).

### 3.2. Educational Levels of Dairy Owners and Awareness of AI Beneficiaries on Timing of AI

In this study, those owners who were illiterate (80.0%) and at a primary level (86.5) of education have inseminated their cows at the wrong time. But those owners who have degree level (28.5%) and secondary level (26.6%) education inseminated their cows at a right time. However, the difference was not statistically significant (*P* > 0.05). If a cow did not conceive for repeated insemination, those who have primary level education (56.6%) used AI again and again, but those who have a degree level of education (66.6%) used natural mating. Regardless of the educational status, most (above 70%) respondents take their cattle to the AI site at the incorrect time after their cattle show heat (Table 2).

The perception of AI beneficiaries' time of insemination depends on the sign of heat in dairy cattle. Therefore, when their cows and heifers showed heat afternoon and morning of the day, 20.5% of them inseminated in the morning of the next day and afternoon that day (right time of insemination), respectively, but the remaining 79.5% inseminated their cows at the wrong time. However, the awareness of the beneficiary was not a statistically significant (*P* value <0.005) constraint of AI service (Table 3).

### 3.3. Major Problems of AI Provision Recognized in Survey Site

AI service problems identified in the study site are conception failure, the shortage of AITS, and the death of calf during the parturition. Among these, conception failure was the most prevailing AI service problem regardless of the kebeles, and it was higher in Ketala (32 (76.2%)) and less in Eta (18 (45.0%)). Death (dystocia) was the least AI service problem in all kebeles. In Ashera and Ketala, the unavailability of the AIT was not a problem. In Ashera, dystocia was not a AI service problem. In the study area, 44 (22.0%) of the respondents have not encountered AI service problem. There was a statistically significant (*P* value <0.05) difference in the identified AI service problems among the study kebeles (Table 4).

#### 3.3.1. Problems of AI Service in Association with the Age and Breed of Cow

In this study, conception failure was highest in adult cows followed by young than by old cows, but the difference was not statistically significant (*P* > 0.05). Dystocia was the same AI service-related problem in all age groups with the same rate, and it has no statistically significant (*P* > 0.05) difference. However, low genetic improvement, low milk production, and low adaptability to disease were not statistically significant (*P* > 0.05) in AI service-related problems (Table 5).

#### 3.3.2. Inbreeding Problem in the Survey Site

The respondents' awareness of the inbreeding issue was highest in Ketala (22 (52.4%)), followed by Mexoma (8 (21.8%)), and the lowest was recorded in Ashera (1 (2.4%)). Among the study kebeles, there was a statistically significant variation in the rate of inbreeding issues (*P* value <0.005) (Table 6).

### 3.4. Detection of Estrus Signs

From total respondents, 98 (48.5%) dairy farmers indicated that bellowing is the most frequent estrus sign they used to detect the heat of their cattle and take them to AI service followed by vulval discharge (23.0%) and mounting on other cows (10.0%) regardless of the kebele. The signs of estrus have revealed a statistically significant difference (*P*=0.002) with AI service (Table 7).

### 3.5. Problems Associated with AI Service among Users Utilizing Communication

About 49.0% communicate with AI technicians through cell phones while half of AI users (50%) get AIT service at the station. These two systems were based on distances between AI service stations, duration of heat detection, and personal agreements. There was no statistically significant difference in the methods the AI beneficiaries communicate to the AITs with AI service (*P* value<0.005) (Table 8).

### 3.6. Overall Satisfaction Assessment of AI Service in the Study Area

In the current study, of 200 dairy cattle owners, 43 (21.5%) were satisfied with the artificial insemination service, and the remaining 157 (78.5%) were not satisfied with the artificial insemination service. There was no statistically significant difference among kebeles (*P* value >0.005) (Table 9).

## 4. Discussion

The present study revealed that about 81.5% of the farmers get regular AI service which is in close agreement with the previous report of Ibrahim and Seid [19] who stated that 85% of small-scale farmers in Western Shoa Zone, Ethiopia, received regular AI service, but higher than the twofolds of prior studies by [20–22] who documented 34.2%, 27.7%, and 30.2% in Wolaita Sodo, Kaliti, and Debretabour, respectively. This disparity might be accounted by farmers' knowledge of estrus and their understanding of the value of crossbreeding. The different accessibility, travel distance from the AI facility to the kebeles, and the proportion of dairy cattle that are present in the kebeles may be the causes of the differences in the lack of inputs among the kebeles. This was greater than the finding of Juneyid et al. [23] who found that 56.3% of small-scale farmers received their services consistently.

The current study's findings indicate that 18.5% of the respondents were unable to access the AI service regularly. Out of them, 4% were denied services owing to the interruption of service on weekends and holidays, 6% were denied services due to a lack of AITs, 1.5% were denied services due to a lack of inputs, 1.5% for all reasons, and 5.5% for unspecified reasons. This finding closely agrees with the finding of Nuraddis et al. [24] who reported that 15% of small-scale dairy cattle farmers do not get regular AI service without interruption but less than half of finding of [23] who documented 43.7% of small scale dairy cattle owners do not get consistent AI service due to the scarcity of AI technicians (18.2%), interruption on weekends and holidays (51.2%) and scarcity of inputs (30.6.0%), shortage of AITs (18%) and interrruption on weekends and holidays (51%) was one of the problems in AI service this was more service in weekends and holidays.

In this study period, the most outstanding constraints of the AI delivery system in the study site were conception failure (62.5%), unavailability of AITs (7.5%), unavailability of AITs and conception failure (4.5%), and dystocia (3.5%). The failure of conception was an increasing constraint in the study area based on the previous trend shown in the report of Temesgen et al. [25] who reported 50.9% of conception failure from the same study area.

This may be attributed to semen quality, improper handling practice, inappropriate time of heat detection and insemination, cow fertility, body condition, and long-distance travel of the cattle for AI service which may result in stress and related health problems and may impact skill of inseminators. This finding corroborates the reports in [2, 26]. The current study's results demonstrate that the farmers were influenced by a scarcity of AIT because of nonuniform distribution of AIT and an upsurge in dairy cattle like the finding of Temesgen et al. [27] who reported that AI technician shortage and technicians' skill level influence the role of AI service in enhancing the genetic performance of the dairy cattle in rural areas and that this issue was both remote and almost negligible.

Artificial insemination (AI) beneficiaries' interpretation of the time of insemination was based on the sign of dairy cattle's heat, but only 20.5% of households are aware of the proper timing of the AI for their cows and receive the service; the remaining 79.5% of households inseminate their cows at the incorrect time. This reveals that wrong timing of the AI by the owners was the influential constraint of AI service in the study area. The effect of wrong timing of the AI on efficiency of AI in the current study was higher than the previous finding of [20] who reported 40.9% of households inseminate their cows at the right time of insemination but, the rest 59.1% of households inseminate their cows in the wrong time and [28] who has shown that 39.8% respondents have inseminated their cows at the right time of insemination but, the rest 60.2% of respondents inseminate their cows at the wrong time.

Regarding the site of the AI service provision, 49.0% of respondents get AIT service at the station, whereas 50.0% get service through cell phones. These two systems were based on the distance between the owner's residence and the AI service station, duration of heat stress, and personal agreements. AI technicians are unable to get transport facilities like motor bicycles and fuel as needed, so farmers must move their cows for long distances in search of AI service. AI is known to be a time-dependent activity, in which during this long journey/waiting time, the heat period is passed away before the service has been given [29].

The survey found that 43 (21.5%) from 200 respondents were delighted with artificial insemination service, whereas 157 (78.5%) were not. This corroborates the finding of Juneyid et al. [23] who reported that 55.8% of users were dissatisfied in various ways when using AI services due to a lack of AI provision on weekends and holidays, AIT shortage, and a limitation of inputs. On the other hand, 172 (44.2%) were satisfied with the AI provision. This demonstrates that most dairy cattle owners do not access AI services appropriately as they want at the correct window of the insemination to improve their animals' genetic potential as well as their productivity.

## 5. Conclusion and Recommendations

The major constraints associated with AI service in the Kacha Bira district were found to be conception failure, unavailability of artificial insemination technicians, dystocia, both conception failure and unavailability of artificial insemination technicians, and AI service interruptions during both regular working hours and weekends and holidays. Major respondents demonstrated that inbreeding was not the problem. Based on assessments of problems associated with artificial insemination services in Kacha Bira district, more than three-fourth of the respondents have not got the AI service regularly and without interruption. Conception failure was the major problem in the study area that must be considered, and the wrong timing of dairy owners also has a great negative effect on the efficiency of AI service in the study site. Mounting on other animals, clear mucus discharge, milk reduction, inappetence, and restlessness are the main heat detection signs used by smallholder dairy farmers. In order to solve the issue and enhance the existing state of conception rate as well as AI efficiency, training and extension programmes should be implemented to promote awareness among animal owners and attendants about the correct timing of the AI and facility and technician-related scarcity should be resolved.

## Figures and Tables

**Table 1:** AI service status in different Kacha Bira district kebeles.

Kebeles	Respondent no.	Get AI service regularly and without interruption (*n* = 163 (81.5%))	Could get AI service consistently (*n* = 37 (18.5%)) due to				
			Lack of AI service during weekends and holidays (%) (*n* = 8 (4%))	Shortage of AITs (%) (*n* = 12 (6%))	Shortage of inputs (%) (1.5%)	All reasons (3 (1.5%))	Other reason (11 (5.5%))
Ashera	41	32 (78.0%)	0 (0%)	4 (90.8%)	0 (0%)	0 (0%)	5 (12.2%)
Mexoma	40	37 (92.5%)	3 (7.5%)	0 (0%)	0 (0%)	0	0 (0%)
Eta	39	28 (71.8%)	0 (0%)	5 (12.82%)	2 (5.1%)	3	1 (2.6%)
Gemesha	38	29 (76.3)	2 (5.3%)	3 (7.9%)	1 (2.6%)	0 (0%)	3 (7.8%)
Ketala	42	37 (88.1%)	3 (7.1%)	0 (0%)	0 (0%0	0 (0%)	2 (4.7%)
*X* ^2^	40.7700						
*P* value	0.004						

**Table 2:** Association of AI services with the level of owner's education.

Variables	Educational level	Degree level *N* (%)	Secondary level *N* (%)	Primary level *N* (%)	Illiterate *N* (%)	*X* ^2^	*P* value
Insemination timing	Inseminate afternoon cow show estrus afternoon (wrong time)	15 (71.4%)	44 (73.3%)	77 (86.52)	24 (80.0%)	4.9933	0.172
	Inseminate in next day cow in estrus afternoon	6 (28.6%)	16 (26.7%)	12 (13.5%)	6 (20.0%)		
	Inseminate morning cow show heat morning (wrong time)	16 (76.2%)	44 (73.3%)	76 (85.4%)	22 (73.3%)	4.0347	0.258
	Inseminate afternoon cow show heat in the morning	5 (23.8%)	16 (26.7%)	13 (14.6%)	82 (6.7%)		

If the cow did not conceive to repeated insemination	Use AI again and again	7 (33.3%)	36 (40.5%)	34 (56%)	16 (53.3%)	5.8291	0.120
	Use NM	14 (66.7%)	53 (59.6%)	26 (43.3%)	14 (46.6%)		
	No *f* (%)	3 (14.3%)	7 (11.7%)	17 (19.1%)	10 (33.3%)		

**Table 3:** Attitude of respondents towards the insemination time in the estrous cycle of their cattle.

	Cows and heifers showed heat in the afternoon		Cows and heifers showed heat in the morning		Total
	Inseminated afternoon	Inseminated the next day morning	Inseminated morning at that time	Inseminated afternoon that day	
Ashera	36 (87.8%)	5 (12.2%)	34 (82.9%)	7 (17.1%)	41
Mexoma	32 (80.0%)	8 (20.0%)	35 (87.5%)	5 (12.5%)	40
Eta	330 (76.9%)	9 (23.1%)	30 (76.9%)	9 (23.1%)	39
Gemesha	28 (73.7%)	10 (26.3%)	25 (65.8%)	13 (34.2%)	38
Ketala	34 (81.0%)	8 (19.1%)	34 (81.0%)	8 (19.1%)	42
Total	160 (80.0%)	40 (20.0%)	158 (79.0%)	42 (21%)	200
*X* ^2^	2.7629		6.3184		
*P* value	0.598		0.177		

*X* ^2^, chi-square.

**Table 4:** Chief constraints of AI provision recognized in survey site.

Constraints of AI service	Kebele					Respondent no. (%)
	Ashera	Mexoma	Eta	Gemesha	Ketala	
Conception failure	16 (39.0%)	29 (72.5%)	18 (45.0%)	30 (79.0%)	32 (76.2%)	125 (62.5%)
Unavailability of AITs	4 (9.5%)	0 (0.0%)	6 (15.0%)	5 (13.2%)	0 (0.0%)	15 (7.5%)
Death/dystocia	0 (0.0%)	3 (7.5%)	2 (5.1%)	1 (2.6%)	1 (2.6%)	7 (3.5%)
Conception failure and unavailability of AITs	1 (2.4%)	1 (2.5%)	3 (7.7%)	1 (2.6%)	3 (7.1%)	9 (4.5%)
Have no problems of AI service (*n* = 44) 22%	20 (45.5%)	7 (15.9%)	10 (22.7%)	1 (2.3%)	6 (13.6%)	44 (22.0%)
Total	41	40	39	38	42	200

*X* ^2^ = 48.0581; *P* ≤ 0.001; AITs, artificial insemination technicians.

**Table 5:** Association of problems of AI service concerning age and breed of cattle.

Problems in AI service	Risk factors	Frequency	Percentage	*X* ^2^	*P* value
	*Age*				

Conception failure	Young	29	14.5	6.5071	0.591
	Adult	63	31.5		
	Old	42	21.0		

Dystocia/death	Young	3	1.5		
	Adult	3	1.5		
	Old	1	0.5		

	*Breed of cow*				

Conception failure	Local	71	35.5	3.7274	0.444
	Exotic	63	31.5		

Dystocia/death	Local	4	2.0		
	Exotic	3	1.5		

Low genetic improvement	Local	7	3.5	4.3327	0.228
	Exotic	13	6.0		
	Total	20	10.0		

Low milk production	Local	14	7.0		
	Exotic	7	3.5		

Low adaptability	Local	2	1.0		
	Exotic	2	1.0		

Have no problem	Local	85	42.5		
	Exotic	70	35.0		

**Table 6:** Problem of inbreeding in the survey site.

Problem	Kebele					Total
	Ashera	Ketala	Mexoma	Gemesha	Eta	
Low genetic improvement	1 (2.4%)	16 (38.1%)	0 (0%)	0 (0%)	3 (7.6%)	20 (10.0%)
Low milk production	0 (0.0%)	6 (14.3%)	8 (20.0%)	3 (7.7%)	4 (10.3%)	21 (10.5%)
Dystocia	0 (0.0%)	0 (0.0%)	0 (0.0%)	1 (2.6%)	3 (7.7%)	4 (2.0%)
No inbreeding problem	40 (97.6%)	20 (47.6%)	32 (80.0%)	34 (89.5%)	29 (74.4%)	155 (77.5%)
Total		42	40	38	39	200

*X* ^2^ = 68.6751; *P* ≤ 0.001.

**Table 7:** Estrus signs respondents used to take their cows for AI service.

Signs of estrus	Kebeles					Total respondents	*X* ^2^ value	*P* value
	Ashera	Ketala	Mexoma	Gemesha	Eta			
Bellowing	17 (41.5%)	23 (54.7%)	23 (57.5%)	22 (57.9%)	12 (30.8%)	98 (48.5%)	43.3102	0.002
Mounting on other cows	4 (9.8%)	5 (11.9%)	0 (0.0%)	5 (13.2%)	6 (15.4%)	20 (10.0%)		
Vulval discharge	15 (36.6%)	7 (16.2%)	13 (32.5%)	5 (13.2%)	6 (15.4%)	46 (23.0%)		
Inappetence	0 (0.0%)	2 (4.8%)	0 (0.0%)	1 (2.6%)	7 (18.0%)	10 (5.0%)		
Milk reduction	2 (4.9%)	1 (2.4%)	4 (10.0%)	3 (7.9%)	5 (12.8%)	15 (7.5%)		
Restlessness	3 (7.3%)	4 (9.5%)	0 (0.0%)	2 (5.3%)	3 (7.7%)	12 (6.0%)		
Total	41	42	40	38	39	200		

**Table 8:** The results of means of communication used to report cows for AI service.

	Kebeles					Total
	Ashera	Mexoma	Eta	Gemesha	Ketala	
AITs visit us daily	1 (2.4%)	0 (0.0%)	0 (0.0%)	0 (0.0%)	1 (2.4%)	2 (1.0%)
We call AITs when we need them	12 (67.3%)	27 (67.5%)	12 (30.8%)	16 (42.1%)	31 (73.8%)	98 (49.0%)
We take our cows to the AI station	28 (68.3%)	13 (32.5%)	27 (69.2%)	22 (57.9%)	10 (23.8%)	100 (50.00%)
Total	41	40	39	38	42	

*X* ^2^ = 30.6347; *P* ≤ 0.001.

**Table 9:** Satisfaction of the respondents with the complete AI services in the study area.

Satisfaction	Kebeles					Total
	Ashera	Ketala	Mexoma	Gemesha	Eta	
Satisfied	7 (17.1%)	6 (14.3%)	8 (20.0%)	8 (21.1%)	14 (35.9%)	43 (21.5%)
Not satisfied	34 (82.93%)	36 (85.71%)	32 (80.0%)	30 (79.0%)	25 (64.1%)	157 (78.5%)
Total	41	42	40	38	39	200 (100%)

*X* ^2^ = 6.6190; *P* ≤ 0.001.

## Data Availability

The datasets used and analyzed during the current study are available from the corresponding author upon reasonable request.

## Abbreviations

AV: Artificial vagina
AI: Artificial insemination
AITs: Artificial insemination technicians
MOE: Ministry of Education
ESAP: Ethiopian Society of Animal Production
IOI: Insemination ovulation time
FAnGR: Farm animal genetic resource
PGF2: Prostaglandin F2 alpha
EAS: Expression analysis systematic explorer.
